# Strengthening research management and support services in sub-Saharan African universities and research institutions

**DOI:** 10.12688/aasopenres.13100.2

**Published:** 2020-11-19

**Authors:** Justin Pulford, Susie Crossman, Sara Begg, Jessica Amegee Quach, Pierre Abomo, Taghreed El Hajj, Imelda Bates

**Affiliations:** 1 Department for International Public Health, Liverpool School of Tropical Medicine, Liverpool, L3 5QA, UK

**Keywords:** Research Management, Research Capacity Strengthening, Capacity Assessment, Sub-Saharan Africa, University, Research Institution

## Abstract

**Background**: International development partners and research councils are increasingly funding research management and support (RMS) capacity strengthening initiatives in sub-Saharan Africa (SSA) as part of a broader investment in strengthening national and regional research systems.  However, the evidence-base to inform RMS capacity strengthening initiatives is limited at present. This research note presents a synthesis of 28 RMS capacity assessments completed in 25 universities/research institutions from across 15 SSA countries between 2014 and 2018.

**Methods**: All 28 capacity assessments were completed following a standardised methodology consisting of semi-structured interviews conducted with research and research support staff at the respective institution as well as document reviews and observation of onsite facilities. Data were extracted from the 28 reports detailing the findings of each assessment according to a framework synthesis approach.

**Results**: In total, 13 distinct capacity gap categories emerged from across the 28 RMS capacity assessment reports.  Almost all the institutions assessed faced multiple gaps in RMS capacity within and across each of these 13 categories. The 13 categories were not independent of each other and were often closely inter-connected. Commonalities were also evident across multiple categories, the two most obvious of which were severe fiscal constraints and the often-complex bureaucracy of the institutional operating environment.

**Conclusions**: The synthesis findings reveal multiple, commonly shared RMS capacity gaps in universities and research institutions across SSA. No single intervention type, or focus, would be sufficient to strengthen capacity across all 13 areas; rather, what is needed to facilitate a significant shift in RMS capacity within such SSA universities and research institutions is a combination of interventions, consisting of differing levels of cost and complexity, variously led (or supported) by both internal and external actors.

## Introduction

Well-developed research management and support (RMS) services ensure a conducive research environment within a university or dedicated research institution. In many sub-Saharan Africa (SSA) countries, RMS capacity is poorly developed ^1, 2^ contributing towards low research production from SSA universities/research institutions relative to their counterparts elsewhere ^3^. International development partners and research councils are increasingly funding RMS capacity strengthening initiatives in SSA settings as part of a broader investment in strengthening national and regional research systems ^4^. However, the evidence-base to inform RMS capacity strengthening initiatives is limited at present ^5^. Large-scale assessments of specific capacity gaps across and between SSA research institutions are scarce and we do not yet have sufficient evidence to reliably inform which types of intervention, in which combinations, with which focus and in what proportion, are required to effectively and sustainably build RMS capacity in SSA settings. Thus, we currently do not understand either what the RMS capacity gaps are or how best to address them.

In this research note, we present a synthesis of 28 RMS capacity assessments completed in 25 universities/research institutions from across 15 SSA countries between 2014 and 2018. Drawing on the findings from this synthesis, we then consider their implications with respect to the design, implementation and evaluation of interventions designed to strengthen RMS capacity in low- and middle-income country settings.

## Methods

The findings presented in this research note have been drawn from a review of 28 project reports. Each report presented the outcome of an RMS capacity assessment completed by the Centre for Capacity Research, Liverpool School of Tropical Medicine (LSTM), in collaboration with the SSA institution being assessed and following a standardised methodology as described elsewhere ^6^. The SSA institutions were collectively participating in eight distinct research capacity strengthening projects and the assessments were conducted in support of their respective programme objectives. Each assessment focused fully or in part on RMS and consisted of semi-structured interviews conducted onsite at the respective institution as well as document reviews (e.g. strategic plans, institutional RMS policies/guidelines, annual reports) and observation of facilities. Interviews and on-site observations were completed by LSTM research staff. The number of interviews per institutional assessment varied (see supplementary file 1, Table 1), ranging between 8–35. A purposive sampling strategy was employed for each assessment, with the aim of obtaining input from staff in positions of strategic interest (e.g. ICT manager) whilst also ensuring participation from a mix of research and research support staff at junior through to senior positions. Pre-visit briefings were conducted remotely with the lead investigator at each institution to explain the purpose and process of the visits and to schedule interviews. Lead investigators were provided with the data collection tools in advance of the visits so they were aware of the range and type of information that would be sought. Interview notes were typed up within a few hours of each interview, checked against audio-recordings of the interviews (available if interviewees gave permission) and final versions verified among the site visit team. Whilst assessments conducted at dedicated research centres tended to span the entire institution, assessments completed at universities typically focused on core RMS services and a focal college or department (e.g. College of Health Sciences or the Department of Public Health). Table 1 (supplementary file 1) lists the country, institution type and focal scientific disciplines (as an indicator of which departments/colleges assessed) of all 28 institutional assessments.

The assessments were designed to gauge the presence and capacity of existing RMS services against an international benchmark. The benchmark was determined based on a review of the RMS literature and in consultation with various stakeholders and focused on six core domains: institutional research strategy; institutional support services; research facilities; human resource management for research; training activities for research; and external promotion of research findings. An exemplar interview guide listing the six RMS domains and the areas examined under each is presented in supplementary file 2. A detailed description of how the benchmark was developed can be found in Wallis *et al.* 2017 ^6^. All assessments were qualitative, with no attempt made to rank or score existing capacities. The assessment was not designed to identify every possible capacity gap as measured against the international benchmark, but rather those capacity gaps that - from the interviewees’ perspective – meaningfully impacted on their ability to conduct or support research. A detailed report (~20–30 pages) describing the identified capacity gaps, strengths, and recommended capacity strengthening actions was completed at the conclusion of each on-site assessment. Reported capacity gaps were based on interviewee comment, document review or observation and were typically based on at least two independent sources to enhance validity (e.g. two interviewees reporting the same challenge or an interviewee reporting a challenge, subsequently verified by observation or document review). Draft reports were shared with representatives from the assessed institution for review prior to finalisation.

Data were extracted from the 28 reports according to a framework synthesis approach ^7^. The framework, constructed in Microsoft Excel, consisted of eight column headings including the institution name, the six core RMS domains listed above and an ‘other’ column and 28 rows, one for each report (see underlying data ^8^). Two independent reviewers, experienced in the institutional capacity assessment process, read the full text of each report and recorded any listed or implied capacity challenges relating to RMS within the corresponding column in the spreadsheet (e.g. ‘unreliable power supply’ would be listed under the ‘research facilities/infrastructure’ column against the respective report). A third reviewer subsequently compared the report extract entries in the spreadsheet. When the same or similar capacity gap was reported by both the initial reviewers, a single representative label was applied to describe it. When a capacity gap was only identified by one of the first two reviewers, the third reviewer consulted the full text of the corresponding report and made a final decision as to its inclusion. Once completed, the recorded entries in the framework were then thematically organised into distinct capacity gap categories. This was an iterative process led by the first author of this research note in collaboration with all co-authors.

## Results

In total, 13 distinct capacity gap categories emerged from across the 28 RMS capacity assessment reports. Each of the 13 categories, along with specific examples of capacity gaps common to each category, are presented in Box 1. Almost all the institutions assessed faced multiple gaps in RMS capacity within and across each of these 13 categories.

Box 1. **Common RMS capacity gaps**

1. Physical Infrastructure
  Unreliable power supply; insufficient laboratory-, office-, study-, meeting or physical storage-space.
2. Information and Communication Technologies (ICT) Infrastructure
  Insufficient ICT hardware; nil/limited access to specialist software; limited internet access or bandwidth capacity.
3. Operating Equipment
  Absence or critical shortage of essential laboratory-, field- and office equipment; vehicle shortage.
4. Laboratory Services and Support
  Poorly maintained laboratory equipment; limited funding to support laboratory maintenance; limited/nil laboratory quality control systems or accreditation; insufficient biosecurity/laboratory safety protocols and resources; nil/sub-optimal revenue generation from provision of laboratory services.
5. Research Funding
  Limited/nil availability of national and/or institutional research funding; limited funding to support post-graduate research required for attainment of award.
6. Workforce
  Excessive workloads for research and research support staff; prolonged staffing vacancies due to hire freezes and/or absence of suitably qualified candidates; aging workforce; under-qualified and/or unexperienced workforce; insufficient laboratory technicians and/or research support staff.
7. Remuneration
  Uncompetitive and/or insufficient salary relative to living costs; inequitable salary ‘top-up’ system applied to externally funded research grants (e.g. academics costed in, but support staff not).
8. Professional Development
  Limited/nil access to training/professional development activities for research and research support staff (technicians and support staff having lowest levels of access); limited/nil institutional structures/services to support professional development; limited/nil staff mentorship schemes; limited/nil staff appraisal and performance mechanisms.
9. Career Progression
  Limited promotion opportunities (especially for technicians and research support staff); job-insecurity; poor staff retention (primarily support staff); limited opportunities for junior academics to enter faculty positions (exacerbated by aging workforce remaining in post).
10. Institutional Support Services
  Inefficient/inadequate financial management-, procurement-, data management-, human resource support services; limited access to research literature/e-resources; limited/nil functionality of institutional review boards.
11. Research Support and Project Management
  Limited/nil pre- and post-award support services, quality assurance and monitoring; limited research cost recovery policies/expertise; limited/nil institutional research strategy.
12. Internal Communication and Collaboration
  Limited internal (inter-departmental) communication and collaboration mechanisms; limited access to and/or awareness of institutional polices and/or available support services.
13. External Communication and Networking
  Limited/nil institutional communications strategy; limited/nil institutional funds and/or staff incentives to support knowledge translation activities; limited/nil research output repository; limited support or oversight of institutional website (content and maintenance).

The 13 categories were not independent of each other, but often closely inter-connected. For example, financial management (i.e. institutional support services) was often constrained by a lack of computing hardware and specialised software (ICT infrastructure), limited training opportunities (professional development), few promotion opportunities (career progression) and perceived low pay (remuneration). Commonalities were also evident across multiple categories, the two most obvious of which were severe fiscal constraints and the often-complex bureaucracy of the institutional operating environment. Many capacity gaps were directly attributable to, or exacerbated by, these two constraints.

## Discussion

The synthesis revealed 13 distinct capacity gap categories, suggesting a diverse array of interventions are needed to ‘shift’ current RMS capacity to a substantially stronger position in universities and research institutions across SSA. Resolving some of the identified capacity gaps would necessitate financial support, for example to purchase required resources (e.g. laboratory equipment or ICT hardware), to invest in high-cost infrastructure developments (e.g. laboratory, study or office space), and to support research funding. In other cases, provision of training or technical assistance (e.g. supporting professional development, laboratory maintenance, development of publication/data depositories) would be more appropriate, and in others, support to strengthen institutional policies, practices and systems (e.g. streamlining and strengthening financial management practices, staff induction and accountability processes, establishing institutional review boards) would be the most relevant action. The extent to which external input, whether from national, regional (within SSA) or international (outside of SSA) sources, is required would vary according to the interventions, ranging from full-to-partial-to nil support. For example, external assistance may be required to support the provision of specialised training or the procurement of otherwise unaffordable equipment, but other interventions could be driven by the respective institutions themselves at a low cost such as the development of remuneration policies or more effective internal communication and collaboration mechanisms.

No single intervention type, or focus, would be sufficient to strengthen capacity across all 13 areas; rather, what is needed to facilitate a meaningful shift in RMS capacity within such SSA universities and research institutions is a combination of interventions, of differing levels of cost and complexity, variously led (or supported) by both internal and external actors. However, interventions that address (even in part) fiscal constraints and complex bureaucracies may be especially impactful given the centrality of these issues across many of the 13 categories reported here. Determining which combination of interventions may be most appropriate for any one institution should be a collaborative process, engaging both research and research support staff (from senior to junior levels) from the focal institution and ideally delivered as part of a longer-term, overarching research capacity strengthening strategic plan. Incorporating robust monitoring and evaluation processes within the capacity strengthening plan would help identify optimal intervention strategies, critical given the paucity of understanding in this area ^5^, and would provide opportunities for shared learning between SSA institutions. Arguably, RMS and broader research capacity within SSA will develop faster the greater the role of regional institutions, Governments and partners in leading the capacity strengthening effort, irrespective of the underlying funding source.

The finding that common capacity gaps existed in many different institutions across multiple countries suggests that time-consuming, external assessments of RMS capacity may not always be required to identify capacity strengthening priorities. Rather, institutional representatives could instead confirm which capacity gaps reported here apply in their context, prioritize these gaps and report additional ones (if any) that might be very specific to their institution and recommend the most appropriate and suitable interventions to mitigate the identified gaps. The commonalities in RMS constraints across institutions further suggests that intervention combinations proven effective be implemented at scale where resources and commitment allow. Finally, not all RMS capacity gaps are equal; thus, assessment processes that allow the relative impact of identified gaps in RMS capacity on subsequent research performance to be better understood would usefully highlight areas for priority intervention in a way that our qualitative approach was not designed to do.

## Data availability

### Underlying data

All requests to the corresponding author for copies of institutional reports will be duly considered. The reports have not been made available as a dataset because the reports cannot be de-identified without compromising anonymity. The reports were produced under ethical approval conditions for the individual projects which stated that only the research team would have access to the data.

Deidentified intermediary data is available from Harvard Dataverse.

Harvard Dataverse: Pulford Justin, Crossman Susie, Begg Sara, Amegee Quach Jessica, Abomo Pierre, El Hajj Taghreed and Bates Imelda, 2020, "Strengthening research management and support services in sub-Saharan African universities and research institutions - anonymous data extraction". https://doi.org/10.7910/DVN/IP3O06 ^8^

This project contains the following underlying data:

- Research Management Systems Challenges Data Extraction - Anonymous.xlsx (Intermediary data extracted from 28 research management system capacity assessment reports)

Harvard Dataverse: Centre for Capacity Research, 2020, "Strengthening research management support services in sub-Saharan African universities and research institutions - table 1: location, institution type, focal science, and number of participants interviewed in each capacity assessment", https://doi.org/10.7910/DVN/HJ6SMJ, Harvard Dataverse, V1 ^9^

This project contains the following underlying data:

- Table 1: Location, institution type, focal science and number of participants interviewed in each capacity assessment

### Extended data

Harvard Dataverse: Centre for Capacity Research, 2020, "Strengthening research management and support services in sub-Saharan African universities and research institutions - example interview guide", https://doi.org/10.7910/DVN/HU5E6Q, Harvard Dataverse, V1 ^10^

This project contains the following extended data:

- An ‘exemplar’ of the interview guides used across the eight projects from which data were drawn to inform the associated research note

Data are available under the terms of the Creative Commons Zero "No rights reserved" data waiver (CC0 1.0 Public domain dedication).
