# Supplementary material for: Strengthening research management and support services in sub-Saharan African universities and research institutions
Source: AAS Open Res. 2020 Nov 19;3:31. Originally published 2020 Jul 20. [Version 2] doi: 10.12688/aasopenres.13100.2 (PMC7780342; doi:10.12688/aasopenres.13100.2)
Supplement: Peer review history

# Peer review: Reviewer response for version 2

**DOI:** 10.21956/aasopenres.14280.r28222

**Published:** 2021-03-01

**Authors:** Aaron N. Yarmoshuk

**Affiliations:** 1 Clinical Public Health, Dalla Lana School of Public Health (DLSPH), University of Toronto, Toronto, ON, Canada

I have now read the revised article in-full and reviewed the responses by the author. I now approve the article without reservation.

Is the work clearly and accurately presented and does it cite the current literature?

Yes

If applicable, is the statistical analysis and its interpretation appropriate?

Not applicable

Are all the source data underlying the results available to ensure full reproducibility?

Partly

Is the study design appropriate and is the work technically sound?

Yes

Are the conclusions drawn adequately supported by the results?

Yes

Are sufficient details of methods and analysis provided to allow replication by others?

Yes

Reviewer Expertise:

Institution Building, Research Management, Capacity Strengthening, Global Health, Globalisation, Global Tertiary Education

I confirm that I have read this submission and believe that I have an appropriate level of expertise to confirm that it is of an acceptable scientific standard.

# Peer review: Reviewer response for version 2

**DOI:** 10.21956/aasopenres.14280.r28221

**Published:** 2020-12-17

**Authors:** Victoria O. Kasprowicz

**Affiliations:** 1 Africa Health Research Institute, Durban, South Africa; 2 Sub-Saharan African Network for TB/HIV Research Excellence (SANTHE), Durban, South Africa

I have no further comments to make. I feel the authors have adequately addressed my concerns.

Is the work clearly and accurately presented and does it cite the current literature?

Yes

If applicable, is the statistical analysis and its interpretation appropriate?

Not applicable

Are all the source data underlying the results available to ensure full reproducibility?

Partly

Is the study design appropriate and is the work technically sound?

Partly

Are the conclusions drawn adequately supported by the results?

Yes

Are sufficient details of methods and analysis provided to allow replication by others?

Partly

Reviewer Expertise:

Immunology, HIV/TB, Research Capacity Strengthening

I confirm that I have read this submission and believe that I have an appropriate level of expertise to confirm that it is of an acceptable scientific standard.

# Peer review: Reviewer response for version 1

**DOI:** 10.21956/aasopenres.14199.r27576

**Published:** 2020-08-12

**Authors:** Aaron N. Yarmoshuk

**Affiliations:** 1 Clinical Public Health, Dalla Lana School of Public Health (DLSPH), University of Toronto, Toronto, ON, Canada

This is a useful research note. It would be good to get a better sense of the 25 institutions where the 28 assessments were conducted, including the 15 SSA countries in which the institutions are based. Fifteen countries represent approximately 1/3 of SSA. Are all sub-regions (Central, Eastern, Southern and Western) represented?  What is the representation between anglophone, francophone and lusophone countries? I can understand why the authors don’t wish to mention specific countries but it would be good to get some idea of the geographic distribution of the 25 institutions.

 “Significant” is used five times in the note yet in the Methods it is stated, “All assessments were qualitative, with no attempt made to rank or score existing capacities.” Without some form of measurement it is suggested that this adjective not be used.

 Are the authors willing to provide the data collection tools used in an appendix? This would be useful for further research. It is noted that the authors provide de-identified intermediary data through the Harvard Dataverse.

 How many representatives were interviewed per institution assessed? Can the range be provided; for example, between x and y representatives were interviewed per institution?

 It is stated that one of the two greatest challenges to strengthening RMS capacity was found to be severe fiscal constraints - was information collected on overhead rates charged by the institutions? If so, can it be presented?

 The other of the two greatest challenges was found to be complex bureaucracy of the institutional operating environment. Is it known if this challenge can be addressed at the institutional level itself or is this partially a creation of having to follow national regulations by which the institutions are governed?

Is the work clearly and accurately presented and does it cite the current literature?

Yes

If applicable, is the statistical analysis and its interpretation appropriate?

Not applicable

Are all the source data underlying the results available to ensure full reproducibility?

Partly

Is the study design appropriate and is the work technically sound?

Yes

Are the conclusions drawn adequately supported by the results?

Yes

Are sufficient details of methods and analysis provided to allow replication by others?

Yes

Reviewer Expertise:

Institution Building, Research Management, Capacity Strengthening, Global Health, Globalisation, Global Tertiary Education

I confirm that I have read this submission and believe that I have an appropriate level of expertise to confirm that it is of an acceptable scientific standard, however I have significant reservations, as outlined above.

# Response

**Published:** 2020-11-10

**Authors:** Susie Crossman

**Affiliations:** Liverpool School of Tropical Medicine, UK

**It would be good to get a better sense of the 25 institutions where the 28 assessments were conducted, including the 15 SSA countries in which the institutions are based. Fifteen countries represent approximately 1/3 of SSA. Are all sub-regions (Central, Eastern, Southern and Western) represented?  What is the representation between anglophone, francophone and lusophone countries? I can understand why the authors don’t wish to mention specific countries but it would be good to get some idea of the geographic distribution of the 25 institutions.**

 Response:  We have included additional information re the country of location for all 28 institutions assessed in a new supplementary file.

**“Significant” is used five times in the note yet in the Methods it is stated, “All assessments were qualitative, with no attempt made to rank or score existing capacities.” Without some form of measurement it is suggested that this adjective not be used.**

 Response: We have replaced the word ‘significant’ with suitable alternatives, less associated with statistical analysis, throughout the manuscript.

**Are the authors willing to provide the data collection tools used in an appendix? This would be useful for further research. It is noted that the authors provide de-identified intermediary data through the Harvard Dataverse.**

 Response: The data collection tools varied somewhat across the eight distinct projects in which the 28 assessments were conducted.  Rather than present the entire suite of data collection tools, we have developed an ‘exemplar’ interview guide listing the six RMS domains and the areas explored under each.  This exemplar interview guide has been uploaded as supplementary file 2. The six RMS domains and their respective question areas were common across all 28 assessments.

**How many representatives were interviewed per institution assessed? Can the range be provided; for example, between x and y representatives were interviewed per institution?**

 Response:  We have included the number of participants interviewed for each assessment in Table 1, supplementary file 1.

**It is stated that one of the two greatest challenges to strengthening RMS capacity was found to be severe fiscal constraints - was information collected on overhead rates charged by the institutions? If so, can it be presented?**

 Response:  Participants were asked whether overhead rates were routinely applied to external grant applications and, if yes, how these rates were calculated and how their application (within grant submissions) was overseen (i.e. who was responsible, how was this monitored). However, we did not record specific overhead rates for each institution assessed, in cases where a standard overhead rate was reported. In general, effective grant costing (including the application of overhead rates) was a common challenge with few participants reporting reliable pre-award support services or processes. This stands out as a critical RMS capacity gap in many institutions given the potential to lose/gain income through poor/robust grant costing practices.

**The other of the two greatest challenges was found to be complex bureaucracy of the institutional operating environment. Is it known if this challenge can be addressed at the institutional level itself or is this partially a creation of having to follow national regulations by which the institutions are governed?**

 Response: Certainly, institutional bureaucracies were often complex due to national regulations over which the respective institutions had limited/nil control.  However, we also observed many examples of bureaucratic procedures that were unnecessarily complex or inefficient and in which the respective institutions could potentially simplify processes.  Common examples were in the areas of financial reporting and procurement where institutional staff were often expending considerable time and effort to complete simple tasks due to a reliance on outdated paper-based systems with onerous and inflexible ‘authorisation/sign-off’ protocols.

# Peer review: Reviewer response for version 1

**DOI:** 10.21956/aasopenres.14199.r27578

**Published:** 2020-08-03

**Authors:** Victoria O. Kasprowicz

**Affiliations:** 1 Africa Health Research Institute, Durban, South Africa; 2 Sub-Saharan African Network for TB/HIV Research Excellence (SANTHE), Durban, South Africa

Pulford *et al.* present results of 28 research management and support (RMS) capacity assessments completed in 25 universities/research institutions from 15 SSA countries performed between 2014-2018. Thirteen distinct capacity gap categories were identified and the authors report that almost all the institutions faced significant gaps both within and across these categories Pulford *et al *.state that these multiple commonly shared gaps could not be strengthened by the introduction on one intervention type, but by a combination of interventions. I enjoyed reading this research note and found it to be a valuable contribution to a relatively limited literature pool. The emerging importance and focus on RMS capacity building highlights the need for further publications in this key area.  

 I acknowledge that this is a short research note but I feel that this publication could be improved with additional information:

- Considering the size of SSA I feel that it would be interesting to state the participating countries and/or more detail with regards to breakdown of institute vs university department. I understand there may be hesitation with listing the specific institutes/universities - but I do feel a little more information could be helpful in interpreting the findings.
- I found the methods/approach a little difficult to fully comprehend with the information provided. For example, the methods stated that an international benchmark was used as a comparison and that this benchmark was determined based on a literature review and consultation with various stakeholders. I think it might be nice to add details on the approach/details of the literature review (e.g. anglophone vs francophone journals, numbers, countries included in the reports etc). Also, it would be of interest to provide details of the participating stakeholders. It would help the interpretation of the paper if more detail on this international benchmark was provided and how it was used. Linked to the benchmark point, and as the authors state ‘significant’ gaps at almost all the sites, I think it might be nice to provide a definition of ‘significant’ and what performance level was identified for the sites who didn’t have significant gaps. 

- I feel it would be beneficial for more detail on the approach at the SSA sites. For example, details on the tools used to help the reader gain a better feel of how capacity gaps were identified e.g. questions for the semi-structured interviews and perhaps a detailed breakdown of the number of participating team members (scientists vs support staff) at each site. Additional questions I have include: What documents were reviewed as part of the assessment? Who carried out the ‘observation of facilities’? How were the documents and observation of facilities used in the process of identifying capacity gaps? How was the research focus/specialty of each site taken into account when identifying specific capacity gaps? How did the capacity gaps actually link with research activity and outputs at each site?

 

 Despite the suggestions noted above, I feel the conclusions that common RMS capacity gaps do exist at many SSA research sites to be useful information that can hopefully guide future interventions efforts. Sites should be encouraged to lead the development of RMS capacity strengthening plans with embedded monitoring and evaluation as part of their strategic plan. Further sharing of gaps, challenges and progress in this area could help identify optimal intervention strategies and opportunities for intra-Africa collaboration.

Is the work clearly and accurately presented and does it cite the current literature?

Yes

If applicable, is the statistical analysis and its interpretation appropriate?

Not applicable

Are all the source data underlying the results available to ensure full reproducibility?

Partly

Is the study design appropriate and is the work technically sound?

Partly

Are the conclusions drawn adequately supported by the results?

Yes

Are sufficient details of methods and analysis provided to allow replication by others?

Partly

Reviewer Expertise:

Immunology, HIV/TB, Research Capacity Strengthening

I confirm that I have read this submission and believe that I have an appropriate level of expertise to confirm that it is of an acceptable scientific standard, however I have significant reservations, as outlined above.

# Response

**Published:** 2020-11-10

**Authors:** Susie Crossman

**Affiliations:** Liverpool School of Tropical Medicine, UK

**Considering the size of SSA I feel that it would be interesting to state the participating countries and/or more detail with regards to breakdown of institute vs university department. I understand there may be hesitation with listing the specific institutes/universities - but I do feel a little more information could be helpful in interpreting the findings.**

 Response: We have now included additional information re the country of location for all 28 institutions assessed in a supplementary file (supplementary file 1).

**I found the methods/approach a little difficult to fully comprehend with the information provided. For example, the methods stated that an international benchmark was used as a comparison and that this benchmark was determined based on a literature review and consultation with various stakeholders. I think it might be nice to add details on the approach/details of the literature review (e.g. anglophone vs francophone journals, numbers, countries included in the reports etc). Also, it would be of interest to provide details of the participating stakeholders. It would help the interpretation of the paper if more detail on this international benchmark was provided and how it was used. Linked to the benchmark point, and as the authors state ‘significant’ gaps at almost all the sites, I think it might be nice to provide a definition of ‘significant’ and what performance level was identified for the sites who didn’t have significant gaps.**

 Response: We sympathise with the request for additional information regarding the development of the international benchmark and other details regarding the capacity assessment methodology (both this comment and below). We have attempted to strike a balance between providing essential detail about the capacity assessment process underlying each of the 28 reports synthesised in this research note, providing essential detail about the methodology employed to synthesise the report content and the need to remain within the limited word count required of the research note format. We have prioritised a description of the synthesis methodology as that, rather than the capacity assessment process, is the primary focus of this research note. However, we acknowledge that the synthesis data reported here cannot be properly understood without a sound understanding of the capacity assessment process underlying the reports.  Fortunately, the latter has been well described in the published literature previously, including a detailed description of the benchmarking process, and we have now clearly signalled in the text where this can be accessed (via open access publication). We recognise this is less than ideal but is perhaps the best solution given the need to balance the various requirements described above.  In addition, we have replaced the word ‘significant’ throughout the research note in order to reduce the perception that we have measured performance by a quantitative means (which we did not) and have made further clarifications around how (lack of) capacity was assessed (see also our responses to reviewer one above).  We have now listed the number of participants interviewed during each assessment in supplementary file 1.  We are not able to provide a reliable breakdown on the percentage of participants in either research or RMS roles, although we have now noted in text that there was generally a relatively even split between the two and have given more detail about the types of participants interviewed.

**I feel it would be beneficial for more detail on the approach at the SSA sites. For example, details on the tools used to help the reader gain a better feel of how capacity gaps were identified e.g. questions for the semi-structured interviews and perhaps a detailed breakdown of the number of participating team members (scientists vs support staff) at each site. Additional questions I have include: What documents were reviewed as part of the assessment? Who carried out the ‘observation of facilities’? How were the documents and observation of facilities used in the process of identifying capacity gaps? How was the research focus/specialty of each site taken into account when identifying specific capacity gaps? How did the capacity gaps actually link with research activity and outputs at each site?**

 Response: We have now included an exemplar interview guide as a supplementary file (supplementary file 2) and have noted participant number in supplementary file one. We have inserted in the text some examples of the types of documents reviewed during the capacity assessments, as well as a description of how the document review/observations were incorporated into the respective institutional capacity assessment reports. We have also more clearly stated who completed the interviews/observations. Assessments were tailored to some degree for each institution, accounting for their respective research focus (or more accurately the focal scientific discipline as reported in Table 1, supplementary file 1), although – in practice – RMS components of the assessment remained largely consistent across all assessments given the relatively generic nature of RMS functions (nb. the assessments usually covered more than just RMS, but only the RMS components are reported in this manuscript). No attempt was made to link documented capacity gaps with research activity/output at the assessed institutions (as also noted in response to reviewer one above). We have more clearly noted this in the text and have included as a recommendation in the discussion.

**Despite the suggestions noted above, I feel the conclusions that common RMS capacity gaps do exist at many SSA research sites to be useful information that can hopefully guide future interventions efforts. Sites should be encouraged to lead the development of RMS capacity strengthening plans with embedded monitoring and evaluation as part of their strategic plan. Further sharing of gaps, challenges and progress in this area could help identify optimal intervention strategies and opportunities for intra-Africa collaboration.**

 Response: Reviewer two was right to point out the key roles of monitoring, evaluation, learning, strategic planning and intra-Africa collaboration in the RMS and broader research capacity strengthening process.  We have amended the discussion accordingly.

# Peer review: Reviewer response for version 1

**DOI:** 10.21956/aasopenres.14199.r27577

**Published:** 2020-07-28

**Authors:** Alex C. Ezeh

**Affiliations:** 1 Dornsife School of Public Health, Drexel University, Philadelphia, PA, USA; 2 School of Public Health, University of the Witwatersrand, Johannesburg, South Africa

- This research note presents and discusses results of 28 research management and support (RMS) capacity assessments of 25 universities and research institutions across 15 countries in SSA. The assessments were implemented between 2014 and 2018. The authors observed that “almost all the institutions assessed faced significant gaps in RMS capacity” across the 13 distinct but inter-related domains they identified. Severe financial constraints and institutional bureaucracy were seen as the two most obvious capacity gaps. The authors concluded that no single intervention would be sufficient to strengthen capacity across all the 13 domains. 
- While this is an important study in an area where very little exists, there are a number of limitations in the current version of the research note. It is not clear how the questions in the semi-structured interview guides were framed. If these sought to identify gaps the respondents saw in their respective institutions, it will not be surprising to come to the conclusion the authors did regarding significant gaps across all the institutions assessed. Respondents will always find answers to any question they are asked in an interview, but such answers may not necessarily provide valid basis for the conclusion on the RMS capacity of these institutions. Even the staff of an institution with the best RMS capacity in the world would find something they could improve on. Stating that there are areas of improvement is not the same as having a capacity gap.
- RMS capacity is not an end in itself; it is supposed to support research. We know nothing about the state of research enterprise at these institutions and the extent to which the identified capacity gaps inhibit research productivity at the institutions. This is particularly important because the nature of what constituted a capacity gap varied enormously in how impactful they could be to research undertaking. For each of the 13 domains, you can almost create a scale that ranges from most severe impact on research to inconsequential impact on research and the inability to distinguish between these types of gaps within a domain in this research note is a major limitation. For example, “unreliable power supply” and “insufficient meeting space” as examples of physical infrastructure gaps are likely to have different impacts on research. There are also variations on how each of the 13 domains could affect research.
- Related to the above point is the issue of the size of these institutions, which could be a university department or college or a whole research institute. To what extent do the identified gaps relate to factors that constraint research at the institutions or aspirational notions of what would be great to have at these institutions? 
- The authors noted that a third reviewer read each of the cases in which a specific gap was identified by only one of the initial two reviewers and then “made a final decision as to its inclusion”. We do not know in how many instances the decision was made to include the gap versus exclude it. Is it possible that the fact that the third reviewer already read about one of the initial reviewer’s assessment of a response as constituting a capacity gap would influence their identification of the gap? This would be the case if in majority of the cases, the third reviewer agreed with the identification of a capacity gap and this would generally exaggerate the number of gaps identified.
- The authors identified a number of the “easy to do” interventions (purchase of laboratory equipment or ICT hardware; investment in laboratory, study or office spaces; training and technical assistance; and strengthening institutional policies, practices and systems). While these are all essential, in the absence of a coherent strategy to strengthen research institutions in SSA, these investments, even if they strengthen RMS at SSA institutions, are unlikely to transform the landscape of research systems in the region. RMS strengthening has to be part of an overall strategy to strengthen knowledge-based institutions in the region. Two critical ingredients to strengthening institutional research capacity in SSA are changing the current funding models and using local African capacity to drive the implementation of any capacity strengthening initiative in the region.
- There are repeated references to the role of external actors in addressing capacity gaps in SSA. This needs further clarification. Current funding models that support SSA institutions through sub-awards and technical assistance from intermediary organizations based outside SSA will continue to undermine the capacity of African institutions. This funding model robs African institutions of access to the levels of funding needed to transform organizational systems and processes, compete for top African talents, and develop closer partnerships with primary funders of research. If it takes capacity to build capacity, the choice of African institutions as primary agencies for capacity building efforts in the region could be transformative. It affirms and further strengthens existing capacity in the region, ensures capacity solutions are appropriate and contextually relevant, and it could guarantee sustained partnerships beyond any specific project or grant.
- I agree with the authors’ final conclusion on the critical role of institutional leaders in defining and prioritizing the capacity gaps/needs of their institutions; and I would hasten to add, and in finding the most appropriate and suitable interventions to mitigate the identified gaps.

Is the work clearly and accurately presented and does it cite the current literature?

Yes

If applicable, is the statistical analysis and its interpretation appropriate?

Not applicable

Are all the source data underlying the results available to ensure full reproducibility?

Partly

Is the study design appropriate and is the work technically sound?

Partly

Are the conclusions drawn adequately supported by the results?

Yes

Are sufficient details of methods and analysis provided to allow replication by others?

Partly

Reviewer Expertise:

Global Health, Population Studies/Demography, Research Capacity Strengthening, Urban Health

I confirm that I have read this submission and believe that I have an appropriate level of expertise to confirm that it is of an acceptable scientific standard, however I have significant reservations, as outlined above.

# Response

**Published:** 2020-11-10

**Authors:** Susie Crossman

**Affiliations:** Liverpool School of Tropical Medicine, UK

**While this is an important study in an area where very little exists, there are a number of limitations in the current version of the research note. It is not clear how the questions in the semi-structured interview guides were framed. If these sought to identify gaps the respondents saw in their respective institutions, it will not be surprising to come to the conclusion the authors did regarding significant gaps across all the institutions assessed. Respondents will always find answers to any question they are asked in an interview, but such answers may not necessarily provide valid basis for the conclusion on the RMS capacity of these institutions. Even the staff of an institution with the best RMS capacity in the world would find something they could improve on. Stating that there are areas of improvement is not the same as having a capacity gap.**

 Response: We agree with reviewer one’s point that there is a distinction between an ‘area for improvement’ and a capacity gap that fundamentally frustrates or undermines the attainment of a sufficient or acceptable level of research capacity. Being qualitative, our assessments were not designed to objectively determine the relative severity of any reported capacity issue on a measurable level of research performance. However, the interviews were designed to identify issues that – from the interviewees’ perspective – meaningfully impacted on their ability to conduct or support research. Where such issues were raised, we also sought to verify these in at least one other interview at the same institute. Thus, whilst the listed capacity gaps vary from major to relatively minor, they were all included in the respective reports from which they were drawn on the basis that they were a genuine cause of frustration for at least one interviewee, but most commonly two or more. To address reviewer one’s concerns, we have now included an exemplar interview guide as a supplementary file (supplementary file 2) and have amended the following sentence in the ‘methodology’ to read (amendment in italics): ‘All assessments were qualitative, with no attempt made to rank or score existing capacities. *The assessment was not designed to identify every possible capacity gap as measured against the international benchmark, but rather those capacity gaps that - from the interviewees’ perspective – meaningfully impacted on their ability to conduct or support research*.’  

**RMS capacity is not an end in itself; it is supposed to support research. We know nothing about the state of research enterprise at these institutions and the extent to which the identified capacity gaps inhibit research productivity at the institutions. This is particularly important because the nature of what constituted a capacity gap varied enormously in how impactful they could be to research undertaking. For each of the 13 domains, you can almost create a scale that ranges from most severe impact on research to inconsequential impact on research and the inability to distinguish between these types of gaps within a domain in this research note is a major limitation. For example, “unreliable power supply” and “insufficient meeting space” as examples of physical infrastructure gaps are likely to have different impacts on research. There are also variations on how each of the 13 domains could affect research.**

 Response: Reviewer one raises an important point and one we fundamentally agree with although, as noted above, our qualitative assessments were not designed to do this.  However, it would be incredibly helpful to the broader research capacity strengthening effort to better understand the relative impact of different RMS capacities on subsequent research performance in the manner described by reviewer 1 and we have now stated this in the final sentence of the discussion.

**Related to the above point is the issue of the size of these institutions, which could be a university department or college or a whole research institute. To what extent do the identified gaps relate to factors that constraint research at the institutions or aspirational notions of what would be great to have at these institutions?**

 Response: As previously noted, the listed capacity gaps were all included in the respective reports from which they were drawn on the basis that – from the interviewees’ perspective – they meaningfully impacted on their ability to conduct or support research. This has been made clearer in the text.

**The authors noted that a third reviewer read each of the cases in which a specific gap was identified by only one of the initial two reviewers and then “made a final decision as to its inclusion”. We do not know in how many instances the decision was made to include the gap versus exclude it. Is it possible that the fact that the third reviewer already read about one of the initial reviewer’s assessment of a response as constituting a capacity gap would influence their identification of the gap? This would be the case if in majority of the cases, the third reviewer agreed with the identification of a capacity gap and this would generally exaggerate the number of gaps identified.**

 Response: The use of independent coders was designed to reduce over- or under-reporting of identified capacity gaps that may result when coding is completed by a single individual.  This method reduces the potential for bias although, as reviewer one notes, some potential for bias remains. We did not keep a record of how often a third opinion was required to make an ‘inclusion/exclusion’ decision; however, a third opinion was only required in a minority of cases and did not always result in a decision to ‘include’ a disputed capacity gap. We further contend that, whilst imperfect, this process is more likely to result in a balanced representation of the report content as compared to a coding process that relied on a single individual.

**The authors identified a number of the “easy to do” interventions (purchase of laboratory equipment or ICT hardware; investment in laboratory, study or office spaces; training and technical assistance; and strengthening institutional policies, practices and systems). While these are all essential, in the absence of a coherent strategy to strengthen research institutions in SSA, these investments, even if they strengthen RMS at SSA institutions, are unlikely to transform the landscape of research systems in the region. RMS strengthening has to be part of an overall strategy to strengthen knowledge-based institutions in the region. Two critical ingredients to strengthening institutional research capacity in SSA are changing the current funding models and using local African capacity to drive the implementation of any capacity strengthening initiative in the region.**

 Response: We are in complete agreement with the views expressed by reviewer one here.  The discussion has been amended to better highlight the important roles of both longer-term strategic planning and regional leadership in research capacity strengthening.

**There are repeated references to the role of external actors in addressing capacity gaps in SSA. This needs further clarification. Current funding models that support SSA institutions through sub-awards and technical assistance from intermediary organizations based outside SSA will continue to undermine the capacity of African institutions. This funding model robs African institutions of access to the levels of funding needed to transform organizational systems and processes, compete for top African talents, and develop closer partnerships with primary funders of research. If it takes capacity to build capacity, the choice of African institutions as primary agencies for capacity building efforts in the region could be transformative. It affirms and further strengthens existing capacity in the region, ensures capacity solutions are appropriate and contextually relevant, and it could guarantee sustained partnerships beyond any specific project or grant.**

 Response: Our use of the term ‘external’ actors refers to persons/organisations external to the focal University or research institution. Thus, an external actor could be national, regional (within SSA) or international (outside of SSA). We have clarified our use of the term in the research note.

**I agree with the authors’ final conclusion on the critical role of institutional leaders in defining and prioritizing the capacity gaps/needs of their institutions; and I would hasten to add, and in finding the most appropriate and suitable interventions to mitigate the identified gaps.**

 Response: We agree and have amended the discussion in line with reviewer one’s comments.
